# Characterization of progenitor cells derived from torn human rotator cuff tendons by gene expression patterns of chondrogenesis, osteogenesis, and adipogenesis

**DOI:** 10.1186/s13018-016-0373-2

**Published:** 2016-03-31

**Authors:** Issei Nagura, Takeshi Kokubu, Yutaka Mifune, Atsuyuki Inui, Fumiaki Takase, Yasuhiro Ueda, Takeshi Kataoka, Masahiro Kurosaka

**Affiliations:** Department of Orthopaedic Surgery, Kobe Rosai Hospital, 4-1-23 Kagoike-dori, Chuo-ku, Kobe, 651-0053 Japan; Department of Orthopaedic Surgery, Kobe University Graduate School of Medicine, 7-5-1 Kusunoki-cho, Chuo-ku, Kobe, 650-0017 Japan

**Keywords:** Human rotator cuff, Multilineage potential, Enthesis, Chondrogenic differentiation

## Abstract

**Background:**

It is important to regenerate the tendon-to-bone interface after rotator cuff repair to prevent re-tears. The cells from torn human rotator cuff were targeted, and their capacity for multilineage differentiation was investigated.

**Methods:**

The edges of the rotator cuff were harvested during arthroscopic rotator cuff repair from nine patients, minced into pieces, and cultured on dishes. Adherent cells were cultured, phenotypically characterized. Then expandability, differentiation potential and gene expression were analyzed.

**Results:**

Flow cytometry revealed that the mesenchymal stem cells (MSC)-related markers CD29, CD44, CD105, and CD166 were positive. However, CD14, CD34, and CD45 were negative. On RT-PCR analyses, the cells showed osteogenic, adipogenic, and chondrogenic potential after 3 weeks of culture under the respective differentiation conditions. In addition, SOX9, type II collagen, and type X collagen expression patterns during chondrogenesis were similar to those of endochondral ossification at the enthesis.

**Conclusions:**

The cells derived from torn human rotator cuff are multipotent mesenchymal stem cells with the ability to undergo multilineage differentiation, suggesting that MSCs form this tissue could be regenerative capacity for potential self-repair.

## Background

Rotator cuff injuries are a commonly encountered cause of shoulder pain and dysfunction. Clinical results of rotator cuff repair have been good for both open and arthroscopic surgery [1, 2]. Despite advances in surgical technique over the past few decades, rotator cuff re-tears occur often after repair. The rate of occurrence of a re-tear is as low as 11 % [3] and as high as 94 % for large and massive rotator cuff repairs [4]. Rotator cuff repair depends on tendon-to-bone healing. In particular, fibrovascular scar tissue forms between the tendon and the bone after surgical repair and fails to regenerate the native enthesis [5]. This scar tissue is weaker than the normal rotator cuff insertion and may make repairs prone to failure [6–8]. In addition, differences in the biological environment at the intra-articular versus extra-articular regions contribute to the histological differences in tendon-bone healing [9]. The synovial fluid, which contains the anti-adhesive protein lubricin, inhibits tendon-to-bone healing [10, 11]. Therefore, insufficient healing is the most frequent complication following surgical reconstruction [3–8]. The ultimate goals of our work are to characterize the human rotator cuff-derived cells, promote regeneration of the native enthesis, and prevent the formation of scar tissue.

Various new biological approaches have been developed to resolve this issue; these include the use of growth factors, bone morphogenetic proteins (BMPs) [12] and, more recently, stem cells [13]. These methods have been used to improve tendon-to-bone healing in vivo. To date, connective tissue progenitor cells are found in many adult tissues, where they are commonly multipotent [14]. Stem cells are originally present in the bone marrow and have been identified as hematopoietic stem cells and mesenchymal stem cells (MSCs). MSCs are multipotent stem cells that can differentiate into cell lineages relevant to orthopedic surgery. Stem cells are in a quiescent state until properly stimulated. When specific programs are applied to the cells in response to stress, they proliferate and differentiate to contribute to the repair mechanism [15]. After rotator cuff repair, the bone marrow cells and tendon-derived cells are involved in repair at the tendon-to-bone interface, and bone marrow cells have been studied frequently. However, only a few reports have characterized human rotator cuff-derived cells. Tenocyte-like cells from the supraspinatus muscle showed expression patterns significantly different from osteoblast and chondrocyte cultures with regard to characteristic markers [16]. In recent studies, MSCs were isolated from human rotator cuff tissue and characterized [17–19]. The native enthesis of the rotator cuff is composed of fibrocartilage layers between the tendon and the bone [20]. However, the chondrogenic differentiation process of human rotator cuff-derived cells remains unclear. Chondrogenic differentiation of human rotator cuff-derived cells has been evaluated at 3 weeks only [18, 19].

In the present study, cells obtained from torn human rotator cuffs were isolated, and their expandability and multilineage potential were analyzed in vitro. The purposes of this study were to investigate whether torn human rotator cuffs have MSC properties and to clarify the chondrogenic, osteogenic, and adipogenic differentiation of human rotator cuff-derived cells.

## Methods

### Isolation of human rotator cuff-derived cells

**Fig. 1:**
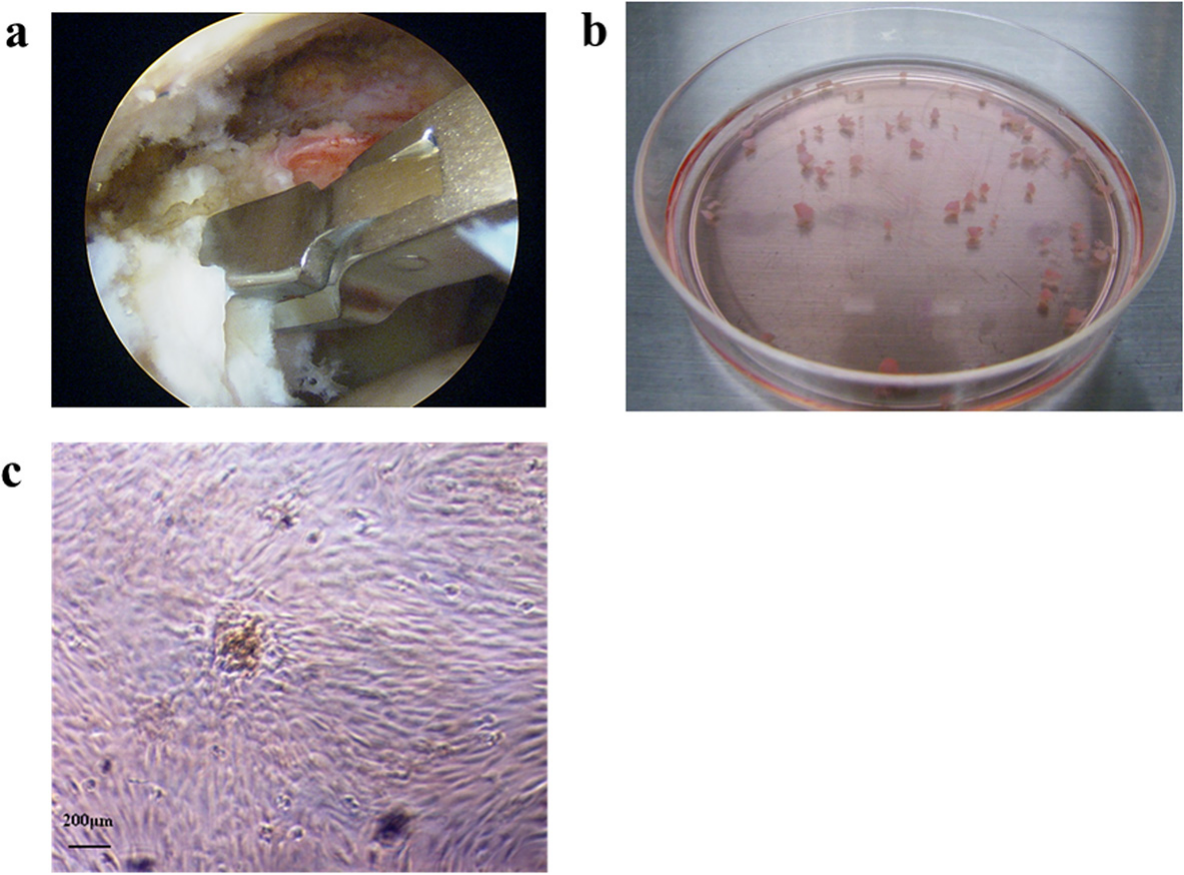
**a** Arthroscopic observations and torn rotator cuff harvesting. **b** Pieces of torn rotator cuff were cultured on 100-mm-diameter culture dishes. **c** Adherent cells displaying fibroblastic morphology in the primary culture

The Kobe University Graduate School of Medicine Ethics Committee approved this study (No. 770), and informed consent was obtained from all patients involved. Patients with inflammatory and infectious diseases were excluded. The edges of the rotator cuff were harvested aseptically from nine patients (five men and four women) who had sustained a rotator cuff tear and underwent arthroscopic rotator cuff repair. The mean age of the patients was 62.9 years (range, 52–70 years). The tear sizes were large in five cases and medium in four cases. An arthroscopic punch was used for harvesting (Fig. 1a). Approximately 3–4 mm of tissue was harvested from the edges of the torn rotator cuff and minced into pieces of approximately 1 mm^3^. The minced tissue was placed on a 100-mm-diameter culture dish and cultured in a monolayer with α-modified minimum essential medium (Sigma, St. Louis, MO, USA) containing 10 % heat-inactivated fetal bovine serum (FBS; Sigma), 2-mM l-glutamine (Gibco, Grand Island, NY, USA), and antibiotics (Fig. 1b). The cultures were incubated at 37 °C with 5 % humidified CO_2_. The minced rotator cuff tissue was removed after a 1-week incubation, and the culture medium was changed after 1-week culture. The nonadherent cells were removed twice per week at a medium change after a 1-week incubation. After 2–3 weeks of culture, the cells were harvested with 0.05 % trypsin-0.02 % EDTA (Wako, Osaka, Japan) and passaged into noncoated 75-cm^2^ culture flasks as passage 1. The population-doubling level was calculated for each subculture using the following equation: population doubling = [log_10_ (N_H_) − log_10_ (N_1_)/log_10_ (2)], where N_1_ is the inoculum number, and N_H_ is the cell harvest number [21]. The calculated population-doubling increase was added to the population-doubling levels of the previous passages to yield the cumulative population-doubling level. As the number of adherent cells could be determined for the first time at passage 1, the cumulative doubling number was calculated first for passage 3.

### Immunophenotyping of cells by flow cytometry

The adherent human rotator cuff-derived cells were evaluated for surface protein expression by flow cytometry. Flow cytometry was performed using FACSCalibur (BD Biosciences, San Diego, CA, USA). The cells were washed with PBS with 3 % FBS after detachment with 0.05 % trypsin-0.02 % EDTA (Wako). Cells (5 × 10^5^) were resuspended in 50 μl of PBS with 3 % FBS and incubated with monoclonal antibodies for the following antigens: CD29, CD34, CD45, CD166 (BD Bioscience), CD14, CD44 (Exalpha Biologicals, Watertown, MA, USA), and CD105 (Ancell, Bayport, MN, USA) for 30 min at 4 °C in the dark. CD29, CD44, CD105, and CD166 are a marker for mesenchymal stem cells, and CD14, CD34, and CD45 are a marker that remains negative for non-hematopoietic-lineage cells [22, 23]. Nonspecific mouse PE-conjugated IgG (BD Bioscience) was substituted as an isotype control. After incubation, the cells were washed twice in PBS with 3 % FBS and resuspended in 500 μl of PBS with 3 % FBS for flow cytometry. Allophycocyanin (APC), fluorescein isothiocyanate (FITC), and R-phycoerythrin (PE) were used for fluorochromes. The fluorescence intensity of the cells was evaluated using a FACSAria instrument, and data were analyzed using FlowJo Software (Tree Star, Ashland, OR, USA). Positive and negative controls of each antibody were also analyzed to ensure antibody-specific cell fluorescence. At least 10,000 list mode events were collected for each sample.

### Osteogenic, adipogenic, and chondrogenic differentiation

Cells from passages 2–3 were used in the following differentiation assays of each sample. Adherent cells were separated into three groups and cultured in the respective differentiation media to induce multilineage differentiation.

An aliquot of 2 × 10^5^ cells from the monolayer was cultured for 3 weeks in medium with 10-nM dexamethasone (Sigma), 10-mM β-glycerophosphate (Sigma), and 50-μg/ml l-ascorbic acid-2-phosphate (Wako) to induce osteogenesis. The culture medium was changed twice per week. After 3 weeks, osteogenic differentiation was evaluated by the mineralized matrix that was stained with Alizarin Red S (Hartman Ledden, Philadelphia, PA, USA). The expressions of the osteogenic-related genes alkaline phosphatase (ALP) and osteopontin were evaluated by reverse transcription-polymerase chain reaction (RT-PCR).

An aliquot of 2 × 10^5^ cells from the monolayer was cultured for 3 weeks in adipogenic medium consisting of low-glucose Dulbecco’s modified Eagle’s medium (DMEM; Sigma) with 1-mM dexamethasone (Sigma), 0.5-mM 3-isobutyl-1-methylxanthine (Sigma), 10-μg/ml insulin (Sigma), 0.2-mM indomethacin (Sigma), and 10 % FBS, to induce adipogenic differentiation. The culture medium was changed twice per week. After 3 weeks, adipogenic differentiation was evaluated by the cellular accumulation of neutral lipid vacuoles that stained with Oil Red O (Muto Pure Chemicals, Osaka, Japan). Adipogenic differentiation was investigated by RT-PCR analysis to detect peroxisome proliferator-activated receptor-γ (PPAR-γ) and lipoprotein lipase (LPL).

A three-dimensional pellet culture was performed for 1, 2, and 3 weeks to induce chondrogenesis. An aliquot of 5.0 × 10^5^ cells in a 15-ml polypropylene tube was centrifuged at 1600 rpm for 4 min to form a pellet. The cells were resuspended in chondrogenic medium consisting of high-glucose DMEM with 10^−7^ M dexamethasone, 50-μg/ml l-ascorbic acid-2-phosphate (Sigma), 0.4-mM proline (Sigma), 1 % ITS^+^1 (Sigma), 500-ng/ml recombinant BMP-6 (Sigma), and 10-ng/ml recombinant transforming growth factor-beta3 (TGF-β3) (R&D Systems, Minneapolis, MN, USA). The culture medium was changed twice per week. After 3 weeks, chondrogenic differentiation was evaluated by toluidine blue staining (Muto Pure Chemicals, Osaka, Japan). The pellets were embedded in paraffin and sectioned for microscopy. The expressions of the chondrogenic-specific genes SOX9, type II collagen, and type X collagen were evaluated at 1, 2, and 3 weeks by RT-PCR.

Total RNA was extracted using an RNeasy Mini Kit (Qiagen, Valencia, CA, USA), according to the manufacturer’s instructions. Approximately 1 μg of total RNA was reverse transcribed from each sample using a High-Capacity cDNA Reverse Transcription Kit (Applied Biosystems, Branchburg, NJ, USA). The converted cDNA samples were amplified by PCR using PCR Taq Gold DNA polymerase (Applied Biosystems). The gene-specific sets are listed in Table 1. PCR amplification was performed using 1 cycle of initial denaturation for 15 min at 95 °C and multiple cycles of 1 min of denaturation at 95 °C, 1 min of annealing at a primer-specific temperature, a 1-min extension at 72 °C, and a 10-min final incubation at 72 °C. Two microliters of cDNA was used for each PCR amplification.

**Table 1:** RT-PCR primer for differentiation-specific gene expression analysis

Primer	Sequences	Annealing temperature	Cycle
ALP	Forward: 5′-CCCAAAGGCTTCTTCTTG-3′	55 °C	35
	Reverse: 5′-CTGGTAGTTGTTGTGAGC-3′		
Osteopontin	Forward: 5′-ACGCCGACCAAGGAAAACTC-3′	60 °C	40
	Reverse: 5′-GTCCATAAACCACACTATCACCTCG-3′		
PPAR-γ	Forward: 5′-TGGGTGAAACTCTGGGAGATTC-3′	60 °C	35
	Reverse: 5′-CATGAGGCTTATTGTAGAGCTG-3′		
LPL	Forward: 5′-GAGATTTCTCTGTATGGCACC-3′	60 °C	30
	Reverse: 5′-CTGCAAATGAGACACTTTCTC-3′		
SOX9	Forward: 5′-AACATGACCTATCCAAGCGC-3′	55 °C	35
	Reverse: 5′-ACGATTCTCCATCATCCTCC-3′		
COL II	Forward: 5′-TCTGCAACATGCAGACTGGC-3′	57 °C	40
	Reverse: 5′-GAAGCAGACAGGCCCTATGT-3′		
COL X	Forward: 5′-GCCCAAGAGGTGCCCCTGGAATAC-3′	57 °C	35
	Reverse: 5′-CCTGAGAAAGAGGAGTGGACATAC-3′		
GAPDH	Forward: 5′-CCACCCATGGCAAATTCCATGGCA-3′	55 °C	30
	Reverse: 5′-TCTAGACGGCAGGTCAGGTCCACC-3′		

Glyceraldehyde-3-phosphate-dehydrogenase (GAPDH) was used as the housekeeping gene to confirm equal mRNA loading.

## Results

**Fig. 2:**
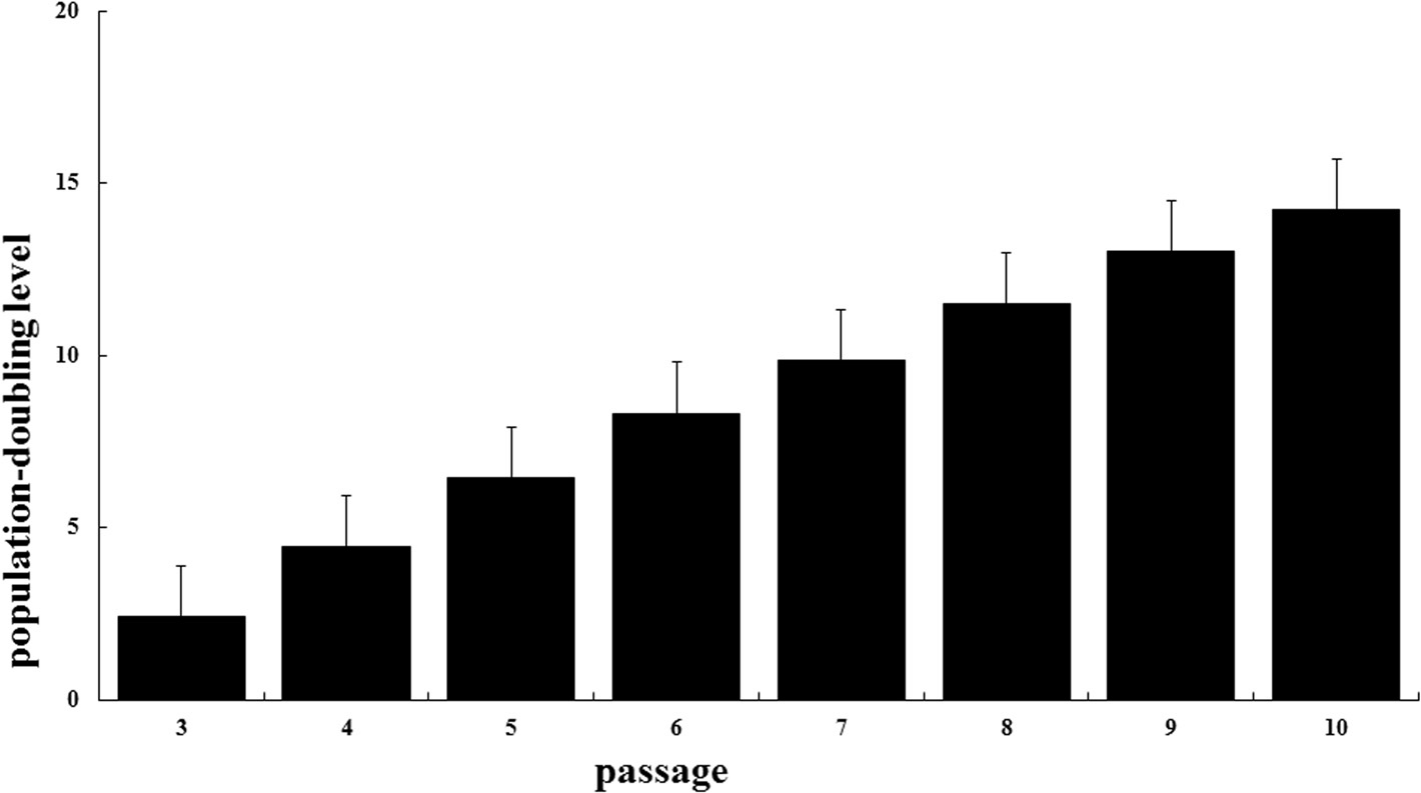
Mean cumulative population doubling values determined at each subculture (*n* = 5)

Adherent human rotator cuff-derived cells began to exhibit a fibroblast-like spindle shape on culture dishes after 1 week. The cells merged and formed a confluent monolayer of fibroblasts (Fig. 1c). These cells could be cultured for at least 10 passages, and 14.2 ± 0.4 population doublings (range, 13.8–15.0), showing neither a change in morphology nor a reduction in proliferation (Fig. 2).

**Fig. 3:**
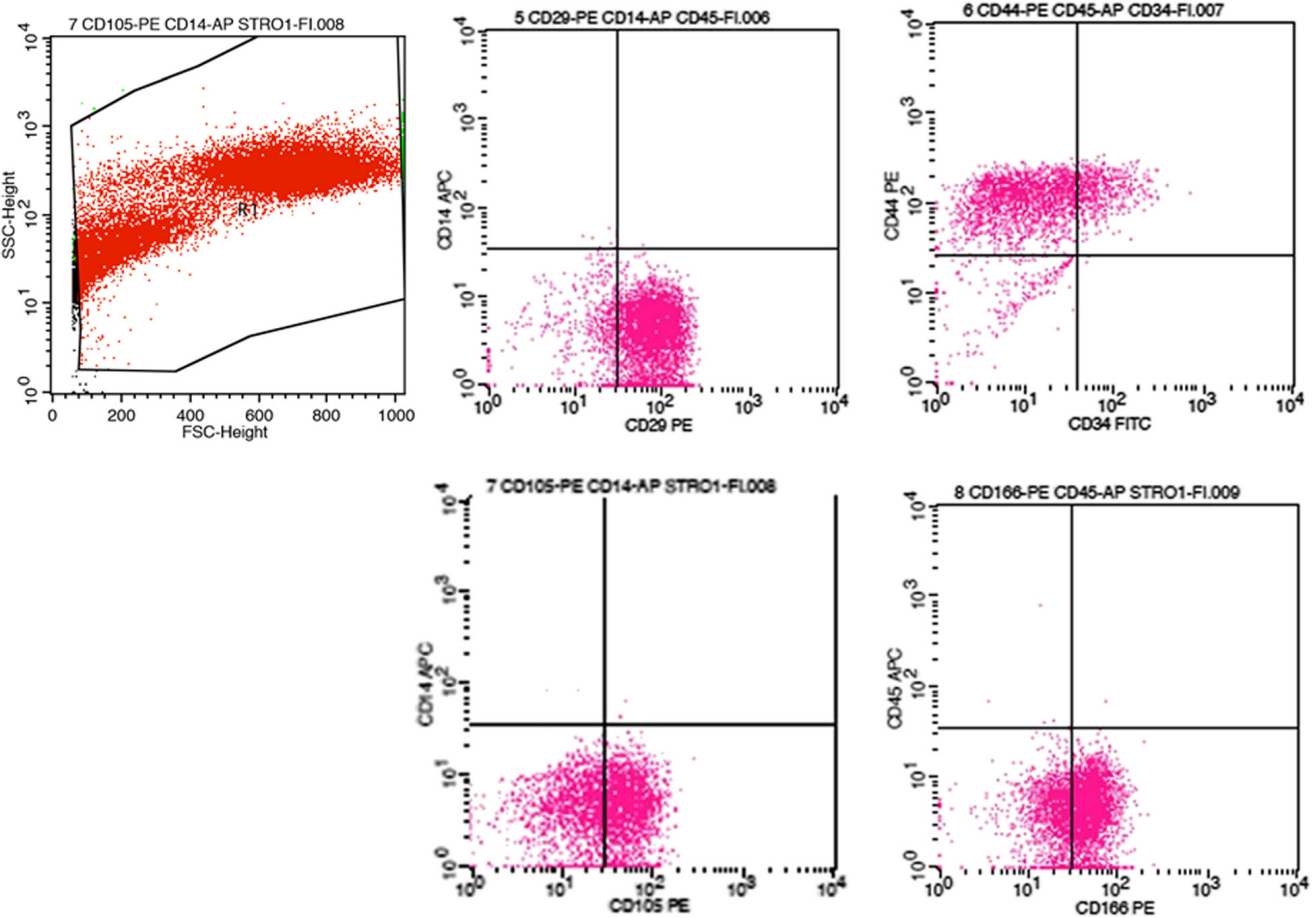
Flow-assisted cell sorting analysis of human rotator cuff-derived cells at the end of passage 1

Adherent human rotator cuff-derived cells at passage 2 were labeled against several cell-surface antigens. On flow cytometric analyses, the cells from the torn human rotator cuff were positive for MSC-related markers CD29, CD44, CD105, and CD166 while negative for hematopoietic-lineage markers CD14, CD34, and CD45. This phenotype is identical to that of MSC. Figure 3 and Table 2 show the cell-surface antigen profile of the human rotator cuff-derived cells.

**Table 2:** Cell-surface antigen expression of the human rotator cuff-derived cells

Cell surface markers	Positive expression rate (%)	Absence (−) or presence (+)
CD14	0.17	(−)
CD29	89.11	(+)
CD34	20.62	(−)
CD44	92.06	(+)
CD45	0.12	(−)
CD105	60.21	(+)
CD166	67.01	(+)

**Fig. 4:**
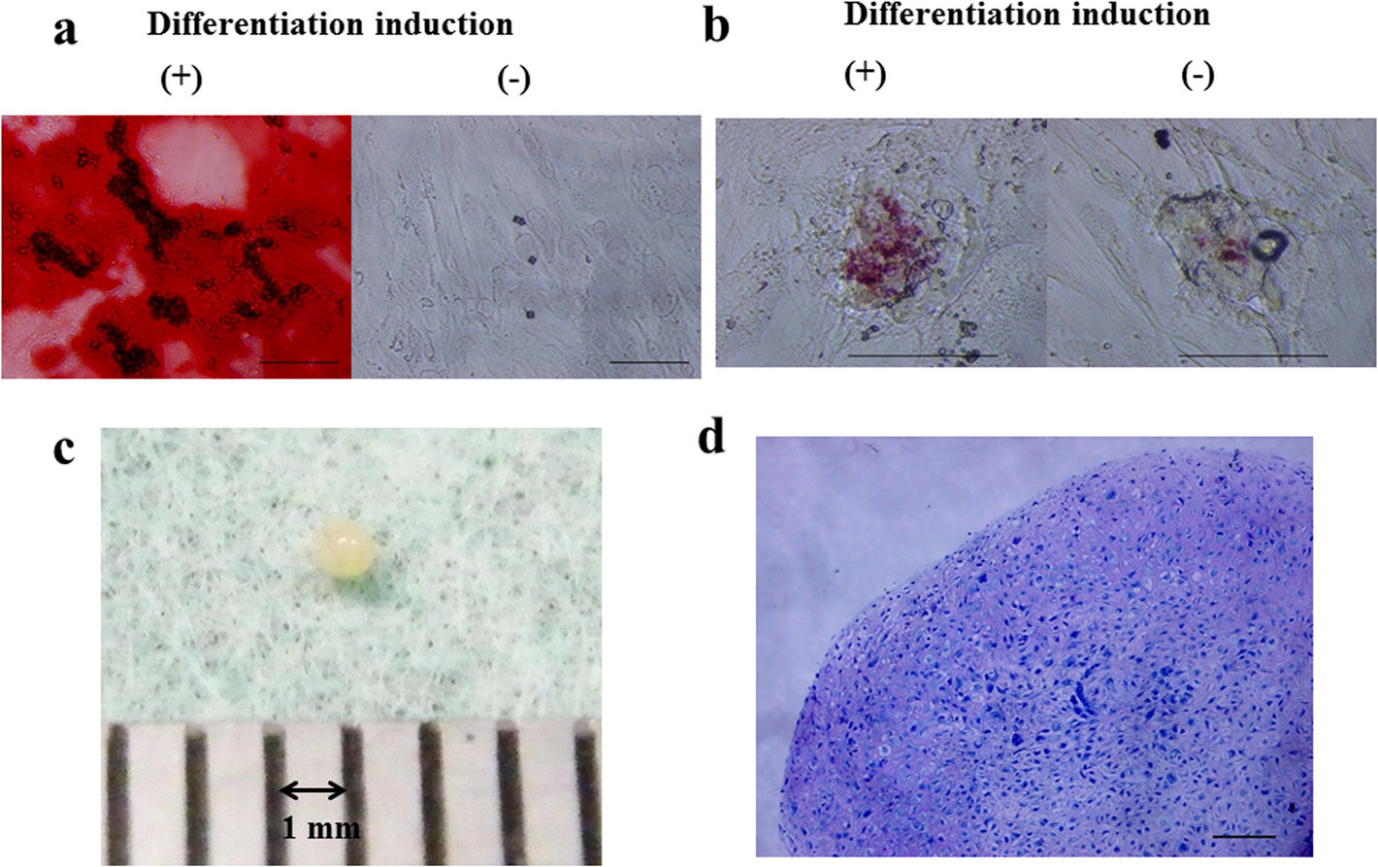
**a** Histochemical analysis of osteogenic differentiation capacity of human rotator cuff-derived cells Alizarin Red S staining after 3 weeks of culture (scale bar 100 μm). **b** Histochemical analysis of adipogenic differentiation capacity of human rotator cuff-derived cells Oil Red O staining after 3 weeks of culture (scale bar 100 μm). **c** Chondrogenic differentiation of human rotator cuff-derived cell pellets. **d** Chondrogenic differentiation capacity of human rotator cuff derived-cell pellets. Histological section stained with toluidine blue after 3 weeks of culture in chondrogenic medium (scale bar 100 μm)

**Fig. 5:**
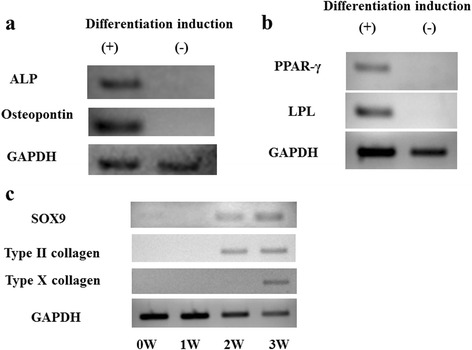
**a** Reverse transcription-polymerase chain reaction analysis of lineage-specific gene mRNA expression in osteogenic culture. The human rotator cuff-derived cells express the osteoblast-related genes alkaline phosphatase (ALP) and osteopontin after 3 weeks of culture under osteogenic conditions. **b** The human rotator cuff-derived cells express the adipocyte-related genes peroxisome proliferator-activated receptor-γ (PPAR-γ) and lipoprotein lipase (LPL) after 3 weeks of culture under adipogenic conditions. **c** The human rotator cuff-derived cells express chondrocyte-related genes such as type X collagen after 3 weeks of culture under chondrogenic conditions. SOX9 and type II collagen expressions are detected after 2 and 3 weeks of culture

Adherent cells were cultured under conditions favorable for osteogenic, adipogenic, or chondrogenic differentiation. After 3-week incubation under osteogenic conditions, adherent cells formed a mineralized matrix as evidenced by Alizarin Red S staining (Fig. 4a). In contrast, no mineralized matrix was observed under undifferentiated conditions at 3 weeks. This osteogenic potential was confirmed by RT-PCR analysis, which showed ALP and osteopontin expression after 3 weeks of culture (Fig. 5a).

Adherent cells formed neutral lipid vacuoles as visualized by Oil Red O staining after a 3-week incubation under adipogenic conditions (Fig. 4b). However, no Oil Red O-positive lipid vacuoles were observed after 3 weeks under undifferentiated conditions. The RT-PCR analysis showed PPAR-γ and LPL expressions under adipogenic conditions after 3 weeks of culture (Fig. 5b). In contrast, no expressions of these genes were observed under undifferentiated conditions.

The pellets had a spherical and glistening transparent appearance after 3-week incubation under chondrogenic conditions (Fig. 4c). The size of the pellets was approximately 1 mm. The cartilage matrix was observed by staining proteoglycans with toluidine blue (Fig. 4d). In the RT-PCR analysis, SOX9, type II collagen, and type X collagen expressions were not observed at 0 or 1 week of culture. However, SOX9 and type II collagen expressions were observed at 2 and 3 weeks of culture. The expression of type X collagen was observed at 3 weeks of culture (Fig. 5c).

## Discussion

Despite improvements in operative treatment for rotator cuff tears, rotator cuff re-tears are still common. Massive rotator cuff repairs can have a failure rate of up to 90 % [4]. This has been largely attributed to degeneration of the rotator cuff tendon. Healing of an injured tendon usually results in formation of poor-quality scar tissue with fatty infiltration and a disorganized matrix [5, 24]. Therefore, it is not surprising that surgical repair of a rotator cuff can lead to high failure rates. Thus, new biological approaches are needed to overcome these problems, such as tissue engineering techniques.

Gulota et al. reported that MSCs alone are insufficient to improve tendon-to-bone healing in animal rotator cuff repair models [13]. The remodeling process for rotator cuff tears remains unclear. The native enthesis of the rotator cuff is composed of four zones, including the tendon, unmineralized fibrocartilage, mineralized fibrocartilage, and bone [20]. The enthesis plays an important role decreasing the mechanical stress between the tendon and the bone. However, fibrovascular scar tissue forms between the tendon and the bone after surgery and fails to regenerate the native enthesis. Tendon-to-bone healing is a major concern after rotator cuff repair, and it is essential to recreate the native enthesis after the surgery. Nourissat et al. reported that MSCs are more effective than chondrocytes for restoring the native fibrocartilage at the enthesis [25]. Therefore, we investigated whether a torn human rotator cuff has MSC properties and demonstrated the chondrogenic differentiation process of rotator cuff-derived cells clearly.

In this study, MSCs from torn human rotator cuffs were isolated and cultured in vitro. The cells were adherent to the plastic culture dishes and were consistently positive for MSC-related surface antigens including CD29, CD44, CD105, and CD166. The human rotator cuff-derived cells also had the capacity for multilineage differentiation, because they differentiated into osteoblasts, adipocytes, and chondrocytes. Therefore, these human rotator cuff-derived cells are MSCs.

Appropriate cellular signals are needed for cells to differentiate into chondrocytes. In this study, human rotator cuff-derived cells cultured with BMP-6 and TGF-β3 differentiated into chondrocytes. TGF-β3 is a growth factor that is unique to fetal development and fetal wound healing. Fetal wound healing occurs without scarring in the presence of high amounts of TGF-β3. It is found during the development of the rotator cuff insertion and is not found during adult tendon-to-bone healing. In addition, TGF-β3 enhances tendon-to-bone healing in a rat rotator cuff model [26]. BMP-6 is a cytokine known to promote chondrogenesis. It was detected in human rotator cuff tissue and was downregulated in calcific tissue [27]. BMP-6 enhances chondrogenesis of the human marrow stromal cells [28]. Therefore, TGF-β3 and BMP-6 may be useful in stimulating a regenerative response to recreate the native enthesis after rotator cuff repair.

SOX9, as the first transcription factor, is essential for chondrocyte differentiation and cartilage formation [29]. In addition, SOX9 is involved in the regulation of collagen 2A1 during chondrogenesis [30]. This was also similar to chondrogenic differentiation of rotator cuff-derived cells. In this study, neither SOX9 nor type II collagen expression was observed at 0 or 1 week of culture, but they were expressed at 2 and 3 weeks of culture. The chondrocyte phenotype is characterized by the expressions of specific genes such as type II collagen and SOX9 [31]. Type II collagen is an abundant component in the cartilage extracellular matrix and is essential for its integrity. SOX9 is involved in chondrogenic differentiation in human rotator cuff-derived cells. Therefore, SOX9 is an essential factor to restore the native enthesis at the tendon-to-bone interface.

Type X collagen has been identified in zones of transition from soft to hard tissue, such as the tendon-to-bone insertion. The expression of type X collagen is absent or reduced during adult tendon-to-bone healing [32]. In the rat Achilles tendon repair model, type X collagen is not detected in the attachment area during the early postoperative period [33]. Type X collagen was detected after the expression of type II collagen prior to fibrocartilage ossification. The repair process of the enthesis is similar to the expression of endochondral ossification [34], which was also similar to the chondrogenic differentiation of rotator cuff-derived cells. In the present study, type X collagen expression was observed under chondrogenic differentiation conditions after 3 weeks of culture. Thus, it appears that it took about 3 weeks for cartilage maturation. These results challenge us to accelerate cartilage maturation in future.

In summary, cells obtained from torn human rotator cuffs were isolated, and their expandability and multilineage potential were analyzed in vitro. The edge of a torn human rotator cuff, which is always discarded, had MSC properties, and the collagen matrix expression pattern during chondrogenesis of the rotator cuff-derived cells was similar to that of endochondral ossification at the enthesis.

The limitations of this study should be discussed. First, because this was an in vitro study, the potential of human rotator cuff-derived cells could not be determined in vivo. Second, the rotator cuff-derived cells were not compared with the bone marrow stromal cells. Further studies are necessary to clarify co-culture of human rotator cuff-derived cells with the bone marrow stromal cells in vitro.

## Conclusions

The torn human rotator cuff-derived cells had multilineage potential in vitro. The chondrogenic differentiation process of torn rotator cuff-derived cells was clearly shown. These cells must play an important role in regenerating the enthesis after rotator cuff repair.
